# Phase stable swept-source optical coherence tomography with active mode-locking laser for contrast enhancements of retinal angiography

**DOI:** 10.1038/s41598-021-95982-9

**Published:** 2021-08-17

**Authors:** Kwan Seob Park, Eunwoo Park, Hwidon Lee, Hyun-Ji Lee, Sang-Won Lee, Tae Joong Eom

**Affiliations:** 1 Advanced Photonics Research Institute, Gwangju Institute of Science and Technology, 123 Cheomdan-gwagiro, Buk-gu, Gwangju, 61005 South Korea; 2 Harvard Medical School, Boston, MA 02115 USA; 3 Wellman Center for Photomedicine, Harvard Medical School and Massachusetts General Hospital, 40 Blossom Street, Boston, MA 02114 USA; 4 Safety Measurement Institute, Korea Research Institute of Standards and Science, 267 Gajeong-ro, Yuseong-gu, Daejeon, 34113 South Korea; 5 Department of Medical Physics, University of Science and Technology, 217 Gajeong-ro, Yuseong-gu, Daejeon, 34113 South Korea

**Keywords:** Biomedical engineering, Applied optics, Medical imaging

## Abstract

Swept-source optical coherence tomography (SS-OCT) is an attractive high-speed imaging technique for retinal angiography. However, conventional swept lasers vary the cavity length of the laser mechanically to tune the output wavelength. This causes sweep-timing jitter and hence low phase stability in OCT angiography. Here, we improve an earlier phase-stabilized, akinetic, SS-OCT angiography (OCTA) method by introducing coherent averaging. We develop an active mode-locking (AML) laser as a high phase-stable akinetic swept source for the OCTA system. The phase stability of the improved system was analyzed, and the effects of coherent averaging were validated using a retina phantom. The effectiveness of the coherent averaging method was further confirmed by comparing coherently and conventionally averaged en face images of human retinal vasculature for their contrast-to-noise ratio, signal-to-noise ratio, and vasculature connectivity. The contrast-to-noise ratio was approximately 1.3 times larger when applying the coherent averaging method in the human retinal experiment. Our coherent averaging method with the high phase-stability AML laser source for OCTA provides a valuable tool for studying healthy and diseased retinas.

## Introduction

Swept-source optical coherence tomography (SS-OCT) is an imaging tool capable of providing three-dimensional information of biological tissues in vivo with high imaging speed, high sensitivity, and high resolution. This desirable performance of SS-OCT is mostly attributable to the laser source performance. SS-OCT uses a wavelength-swept laser as the light source; the wavelength sweep rate and instantaneous linewidth of the laser used in the imaging system determine the imaging speed and imaging depth of the system, respectively. Nonlinear wavelength sweeping and broad instantaneous linewidth can cause a roll-off characteristic (sensitivity decrease in depth) in the system, thereby deteriorating image quality^1^. Till date, several types of wavelength-swept lasers have been developed to improve the speed and depth of SS-OCT imaging; these lasers are based on the Fourier-domain mode-locking (FDML) laser with a Fabry–Perot tunable filter^2^ or polygonal scanning mirror^3^ and a microcavity tunable laser with microelectromechanical systems (MEMS)^4^ depending on the wavelength sweeping and lasing methods. All these lasers involve mechanical movement to sweep the wavelength in the time domain, causing sweep timing jitter and hence low phase stability in SS-OCT. They also require the use of postprocessing, such as k-linearization, to compensate for the nonlinear sweeping of wavelength in time.

Alternatively, different types of wavelength-swept lasers have been introduced to eliminate mechanical movements in the wavelength tuning of the laser, and these are referred to as akinetic wavelength-swept lasers. An akinetic wavelength-swept laser allows high sweeping repeatability and linear sweeping. Consequently, it produces a stable phase in the OCT signals. The recently commercialized akinetic wavelength-swept laser involves an integrated semiconductor optoelectronic design method that does not require mechanical moving parts for wavelength sweeping^5^; it shows excellent system roll-off characteristics without wavenumber domain (k) linearization (postprocessing step). This akinetic laser source is capable of linear wavelength sweeping in the 1060 nm, 1310 nm, and 1550 nm wavelength bands for biological tissue imaging; further, these characteristics have been demonstrated by OCT imaging of the human retina, skin, tooth, and face as well as mouse brain^5–9^. In addition, these lasers have excellent phase stability and offer advantages, such as large coherence length and negligible sensitivity roll-off, as light sources for swept-source OCT angiography (OCTA) systems^7–10^. However, akinetic lasers digitally tune the wavelengths and require high-precision synchronization with the data acquisition device.

An active mode-locking (AML) laser, which is a type of akinetic light source, has also been developed^11, 12^. The AML laser sweeps the wavelengths through dispersion variation using the dispersive material in the AML laser cavity. This material induces chromatic dispersion and causes different round-trip times for beams of different wavelengths in the cavity. At this time, by changing the intensity modulation frequency inside the cavity by applying an external radio frequency (RF) synthesizer signal, the modulation and round-trip frequencies for a specific wavelength are actively mode locked. Thus, only the locked lasing light of a specific wavelength exits the cavity, and the other beams are excluded from the locking condition. Wavelength tuning is performed by full electrical modulation, and this output light has high phase stability. In addition, electric modulation enables programmable wavelength sweeping (linear sweeping in time) so that the axial resolution does not change with depth without a k-linearization procedure. Despite these advantages, there are disadvantages such as shortening of the coherence length owing to the large cavity length in the laser system and deteriorating sensitivity roll-off characteristic of OCT^11–13^. To overcome these limitations, Shirahata et al*.* improved the imaging depth of the OCT system by applying a full-range technique using the high phase stability characteristics of the AML laser^14^. Further, in our previous study, we succeeded in increasing the imaging depth to 2.5 mm by improving the roll-off characteristics with an AML laser of 1080 nm via system optimization (applying high RF modulation frequency and pulse modulation) at a 100 kHz repetition swept rate for successful post-retinal imaging^15^. However, there are no known reports on the characteristics of swept-source AML lasers for enhancing the performance of the OCTA imaging system.

Averaging a set of sinusoidal signals over time in the same phase is called coherent averaging. We use complex rather than absolute values of the measured OCT interference signal to maximize the random characteristics of the complex-valued noise term. Coherent averaging causes the noise term in a set of noisy sinusoidal signals to approach zero, leaving the sinusoidal signals and increasing the signal-to-noise ratio. In previous research, this effect has been used to increase the dynamic range of an OCT system by lowering the noise floor of the OCT signal. However, it has not been studied in the context of OCTA, which calculates the dynamic signal from OCT signals. The OCT signal of the retina-vessel region has temporal random properties, caused by blood-flow. When we perform OCTA by the speckle variance method, the coherence average of the blood flow region will be minimized, and the difference between each OCT signal and the averaged value will be maximized.

In this paper, we present the AML laser-based SS-OCTA system that uses a coherent averaging method for imaging human retinal vasculature. By taking advantage of the characteristics of an AML laser with a stable phase, the coherent averaging method is applied to OCTA, and the improvement in the contrast of the OCTA image is shown. First, we show the performance of the AML laser in terms of phase stability by measuring the standard deviation of the instantaneous phase to verify the phase-stable characteristics without any other phase correction (compensation for timing jitter). Then, the contrast-to-noise ratio (CNR), signal-to-noise ratio (SNR), and vessel connectivity of the OCTA image for a retina phantom were assessed to demonstrate the coherent averaging effect in OCTA. Finally, human retinal vasculature was imaged, and the contrast values of the OCTA images acquired with and without coherent averaging were compared, similar to those in the phantom experiment.

AML laser-based OCTA system for imaging retinal vasculature has been verified for the first time through a retina phantom and in vivo human retinal imaging. Further, we found that the CNR of the OCTA image can be improved by 1.3 times by introducing coherent averaging method.

## Results

### Measurement of the phase stability of the AML laser

**Figure 1:**
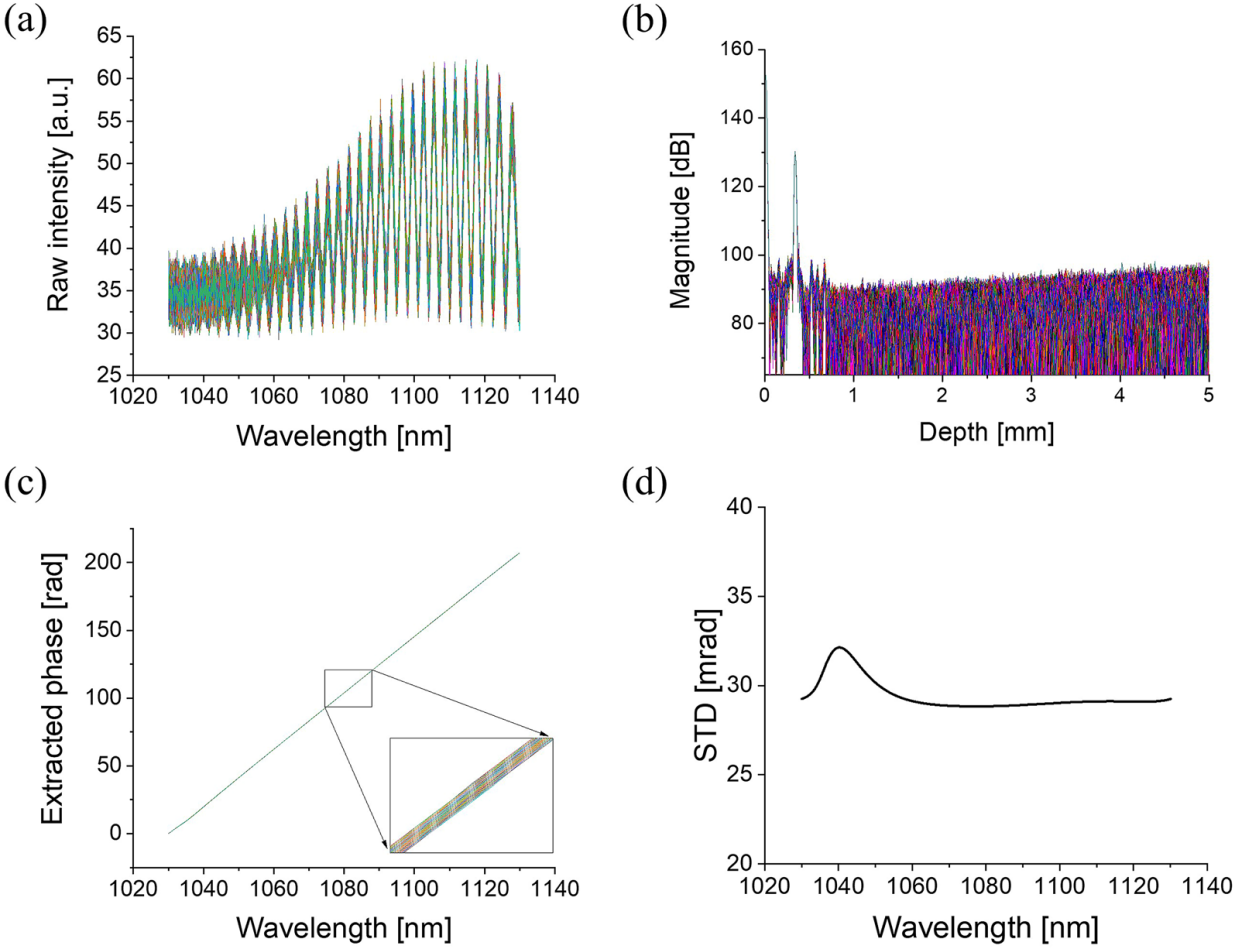
Wavelength sweep repeatability of the active mode-locking laser used in experiments. (**a**) Measured interference signals with the cover glass. (**b**) Inverse fast Fourier transform of the measured signals (**a**). (**c**) Phase obtained by fast Fourier transform of the peak in (**b**). (**d**) Standard deviation of the phase signals of (**c**).

Interference signals measured from a cover glass of thickness 330 μm were analyzed to determine the wavelength-sweeping repeatability of the AML laser. Because the measured signal is the interference of light beams reflected from the front and back of the cover glass (common path), it will contain only the phase fluctuations caused by the wavelength-swept laser and exclude the system noise. Figure 1a shows the measured raw spectra in which a total of 1600 consecutive A-line signals are superimposed. After obtaining the inverse fast Fourier transforms of the signals, signals with a single dominant peak are obtained, as shown in Fig. 1b. Thereafter, we zeroed all values other than those in a seven-pixel range around the center with the strongest signal and take the Hanning window to reduce computational artifacts caused by the Gibbs phenomenon, then repeated the fast Fourier transform (FFT); these results are shown in Fig. 1c, where the inset shows the phase shift for each wavelength. The signal in the seven-pixel range covers the signal level of − 40 dB from maximum peak level. To determine the amount of phase fluctuation between each pair of consecutive A-line signals, the standard deviations (STD) of the 1600 A-lines were obtained for each wavelength (Fig. 1d). The STD was found to be approximately 30 mrad for all wavelength. However, at 1040 nm, the measured STD value was 40 mrad; this is somewhat higher than the STDs at the other wavelengths and appears to have added phase noise due to the weak signal strength.

### Correction of phase differences between the B-scans

**Figure 2:**
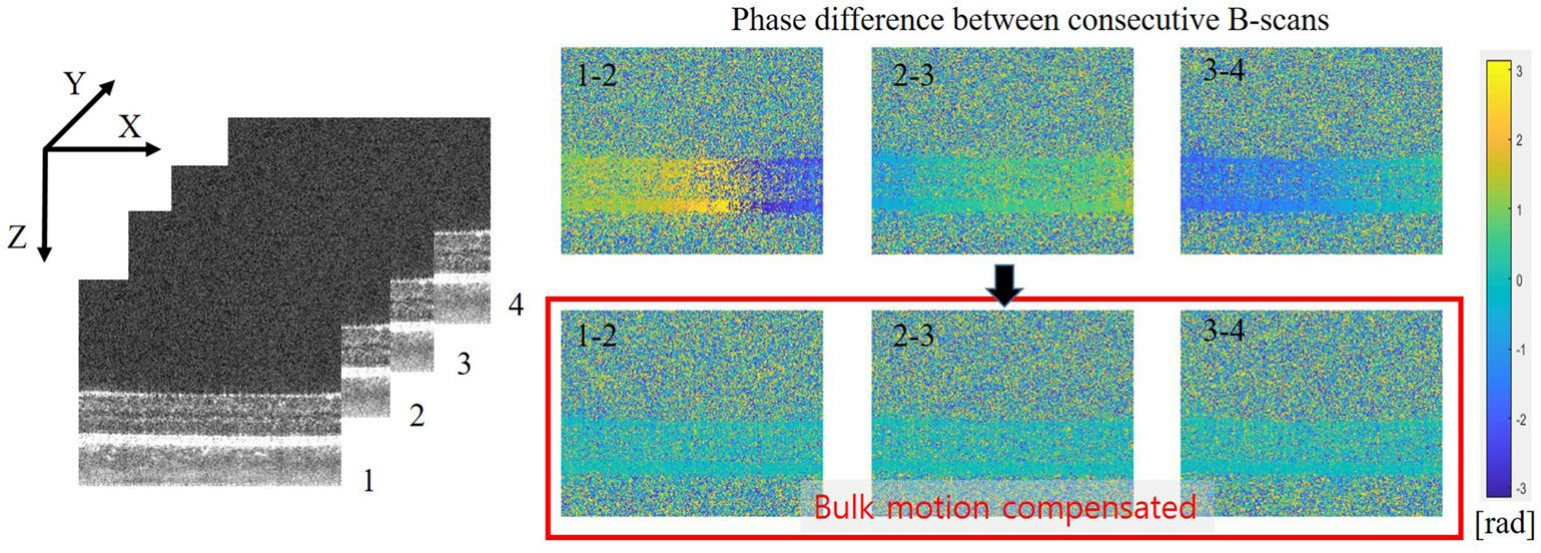
Phase difference between consecutive B-scans (top) before and (bottom) after compensation for bulk motion of the subject. Numbers 1, 2, 3, and 4 indicate consequently acquired B-scans.

To improve the contrast of an OCTA image, it is important to remove artifacts caused by motion. In this study, the phase differences between consecutive B-scans due to bulk motion were removed before computing the OCTA image. Figure 2 shows the phase differences between four consecutive B-scans acquired at the same C-scan location. It was observed that the phase shift introduced by the axial motion. By calculating the cross-correlations between each consecutive pair of B-scans, the phase differences between the consecutive frames were corrected so that they were close to zero, as shown in the bottom row of Fig. 2.

### Validation of coherent averaging effect in OCT with retina phantom

The human eye is capable of different types of voluntary movements, and the resulting motion artifacts cause blurring and loss of contrast in the OCT images. To avoid such ambiguities and determine the coherent averaging effects on the OCT/OCTA images, we imaged a retina phantom before imaging the human retina with the developed AML laser-based OCTA system. The retina phantom, which mimics the retinal layers and blood vessels to enable evaluation of the retinal OCTA performance, comprised an appropriate amount of a scatterer (tissue) and microfluidic channels (vasculature) to simulate the human retina. A detailed description of the retina phantom is presented in the “Methods” section. An intralipid solution of concentration 2% was injected instead of blood into the microfluidic channel of the phantom from the outside using a syringe with an inner diameter of 5.585 mm. The injection rate was controlled at a constant value of 0.03 mL/min using a syringe pump.

To explore the effects of coherent averaging, we obtained OCT images by applying magnitude and coherent averaging. The magnitude-averaged OCT signal, ${\mathrm{I}}_{magAvg}$, is given by.

1 $${\mathrm{I}}_{magAvg} = \frac{1}{N}\sum_{k=1}^{N}\left|{C}_{ijk}\right|,$$

and the coherent-averaged OCT signal, ${\mathrm{I}}_{cohAvg}$, by

2 $${\mathrm{I}}_{cohAvg} = \left|\frac{1}{N}\sum_{k=1}^{N}{C}_{ijk}\right|.$$

In Eqs. (1) and (2), *C* is the complex signal obtained from the FFT of the interference signal; indices *i* and *j* represent the pixel numbers along the A-scan and B-scan, respectively; and *k* represents the number of repetitions the B-scans for a given C-scan location.

**Figure 3:**
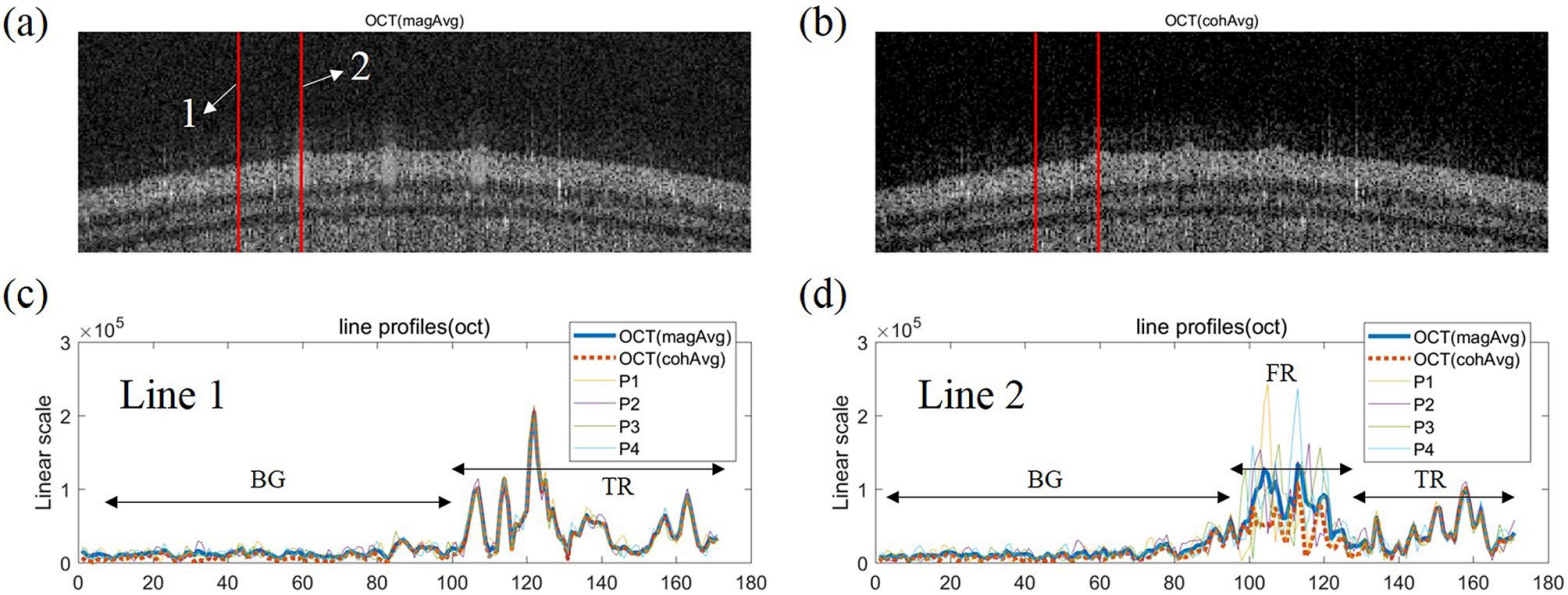
Comparison of OCT images for retina phantom obtained by magnitude averaging (magAvg) or coherent averaging (cohAvg). (**a**) OCT image applying magAvg. (**b**) OCT image applying cohAvg. (**c**,**d**) Profiles of lines number 1 and 2 drawn in the averaged OCT images (**a**,**b**) and four OCT images (P1–4) before averaging, respectively. BG, background region. *TR* tissue region, *FR* flow region.

The results of applying the magnitude averaging method (magAvg) to the magnitude signals of four images and the coherent averaging method (cohAvg) to the complex signals are shown in Fig. 3a,b, respectively. To visualize the differences in the OCT images averaged by these two methods in detail, the line profiles at the positions marked with red lines 1 and 2 in Fig. 3a,b are shown in Fig. 3c,d, respectively; they were obtained from four OCT images (Supplementary Fig. S1) before averaging and are plotted in linear scale to show the differences between them more clearly. Figure 3c shows the line profiles corresponding to the red line 1 and includes signals from the background (BG) and tissue (TR) regions of the phantom.

In the TR spectra of Fig. 3c,d, it can be seen that there is not much difference in the intensity for all four images P1–4, magAvg, and cohAvg. However, in the flow region (FR) of Fig. 3d, the intensity average by magAvg converges to the mean value of those of P1–4 and the average by cohAvg converges to the smallest value from among P1–4. The trend in the BG is identical to that in the FR, as shown in Fig. 3c,d. Therefore, when cohAvg was applied to an OCT image, the background level was lower than that when magAvg was applied, and the SNR of the OCT image could be increased. This result is consistent with previously published results^16, 17^.

### Validation of coherent averaging effect in OCTA with retina phantom

The OCTA calculation by magAvg or cohAvg can be expressed using the following equation by inserting Eqs. (1) and (2) into the speckle variance algorithm^18^.

3 $${OCTA}_{magAvg\, or\, cohAvg} =\frac{1}{N}\sum_{k=1}^{N}{\left(\left|{C}_{ijk}\right|-({I}_{magAvg}\, or\, {I}_{cohAvg})\right)}^{2}$$

**Figure 4:**
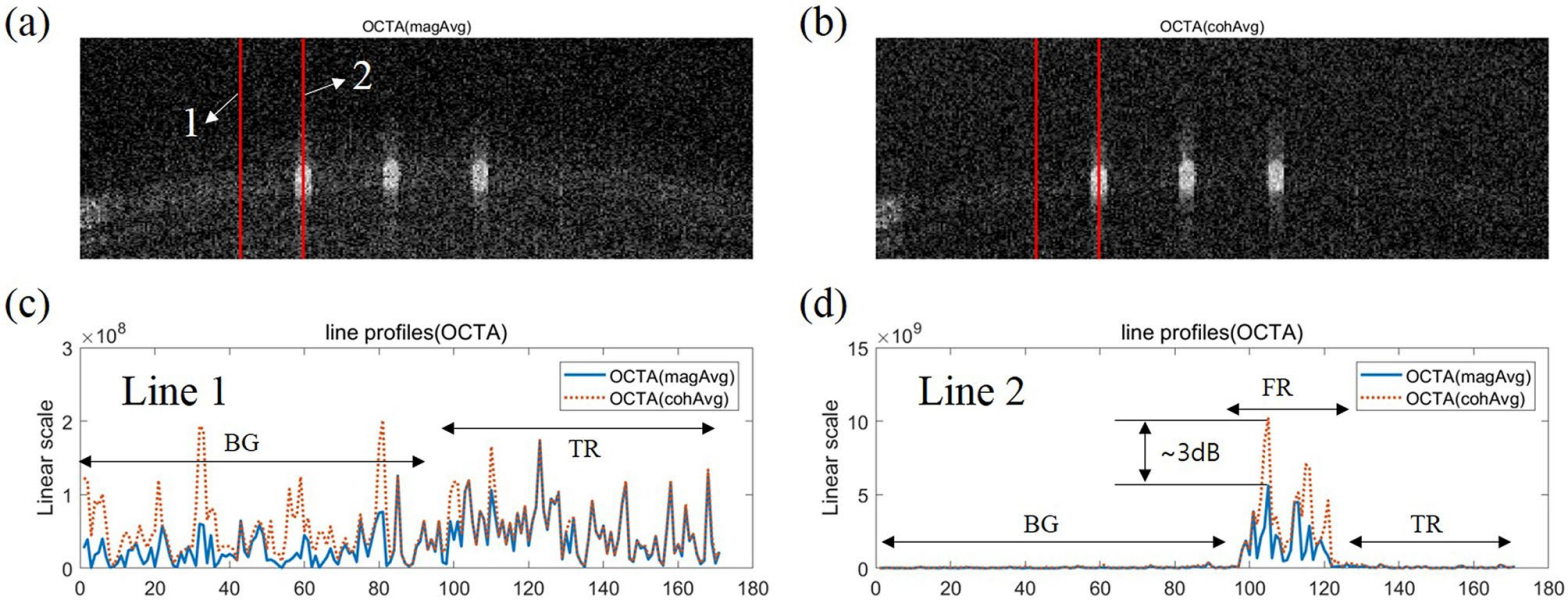
Comparison of OCTA images for retina phantom obtained by magnitude averaging (magAvg) or coherent averaging (cohAvg). (**a**) OCTA image applying magAvg. (**b**) OCTA image applying cohAvg. (**c**,**d**) Profiles of lines number 1 and 2 drawn in the averaged OCTA images, respectively. BG, background region. *TR* tissue region, *FR* flow region.

The general speckle variance algorithm was renamed as OCTA with magAvg. However, the speckle variance algorithm that does not ignore phase changes was renamed OCTA with cohAvg. From the results of the comparative experiments on OCT image averaging shown above, it can be inferred that the variance of the four signals increases in the case of cohAvg compared to that in the case of magAvg. This increase in variance can double the intensity of the signal in the FR. Figure 4a,b represents OCTA images obtained using the speckle variance algorithm as well as by applying magAvg and cohAvg, respectively. The line profiles corresponding to red lines 1 and 2 in Fig. 4a,b are shown in Fig. 4c,d. Unlike the averaged OCT image, the FR and BG signals in the OCTA image were higher in cohAvg than magAvg (Fig. 4d), and the signal in the TR had similar intensity levels for the magAvg and cohAvg (Fig. 4c,d). Although both the FR and BG signals have large variances under cohAvg, the OCTA image contrast is improved because the increase in the variance of the FR signal far exceeds that of the BG signal.

**Figure 5:**
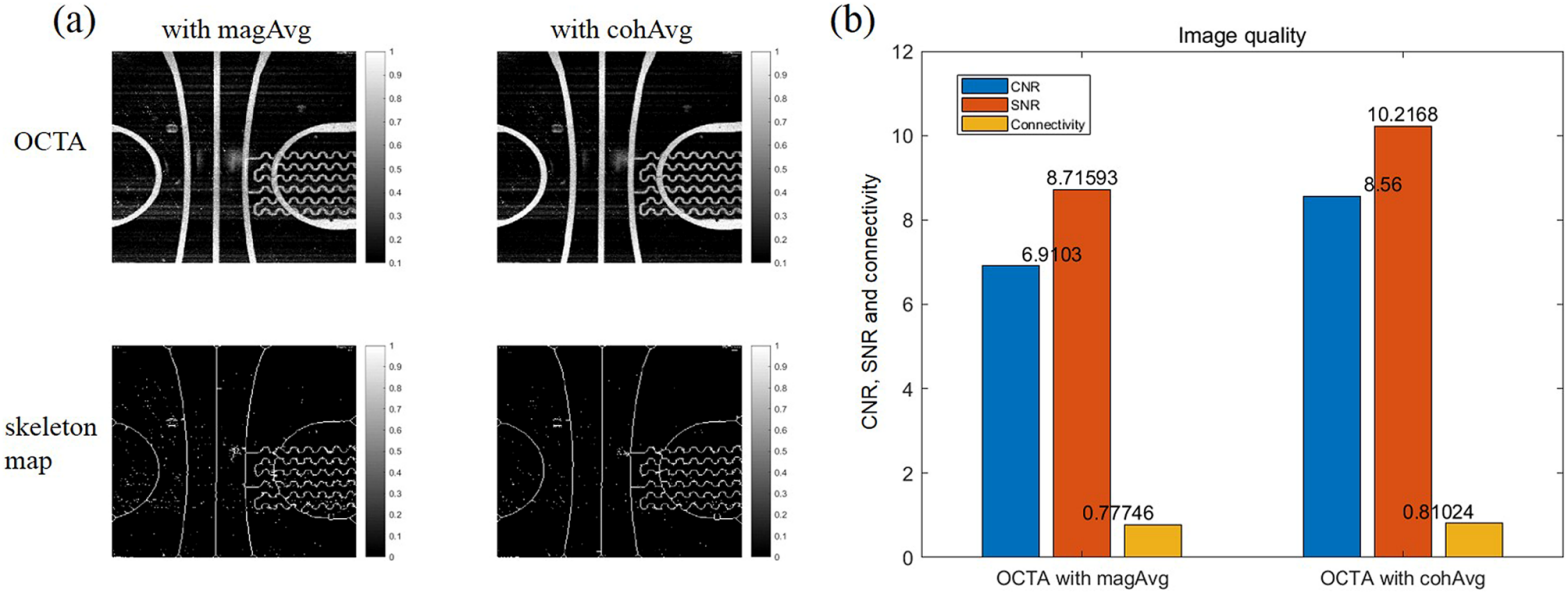
Assessment of en face OCTA images for retina phantom obtained by magnitude averaging (magAvg) or coherent averaging (cohAvg). (**a**) En face OCTA images with magAvg and cohAvg (top row) and their skeleton maps (bottom row). (**b**) Contrast-to-noise ratio (CNR), signal-to-noise raito (SNR), and vessel connectivity.

In the above results, when cohAvg was applied to acquire the OCTA image, both the FR and BG signal levels increased, so it was difficult to determine whether the contrast of the OCTA image increased or decreased in the en face view. For clarity, to evaluate the quality of the en face OCTA image, we assessed the CNR, SNR, and connectivity after layer segmentation with a specific depth range including the flow signal and after performing a maximum intensity projection (MIP) (Fig. 5a). Then, each en face OCTA image was normalized and binarized by applying a threshold value of 0.5 to separate the signal and background regions; the image was subsequently skeletonized, and the CNRs, SNRs, and connectivities of the magAvg and cohAvg images were calculated (Fig. 5b). Detailed descriptions of each of the evaluation indexes are provided in the quantification of en face OCTA images subsection of the “Methods” section. The CNR, SNR, and connectivity of the OCTA image with cohAvg application were 8.56, 10.2168, and 0.81024, respectively, which were higher than the values of the OCTA image with magAvg application, i.e., 6.9103, 8.71593, and 0.77746, respectively.

### In vivo human retinal imaging with coherent averaging

**Figure 6:**
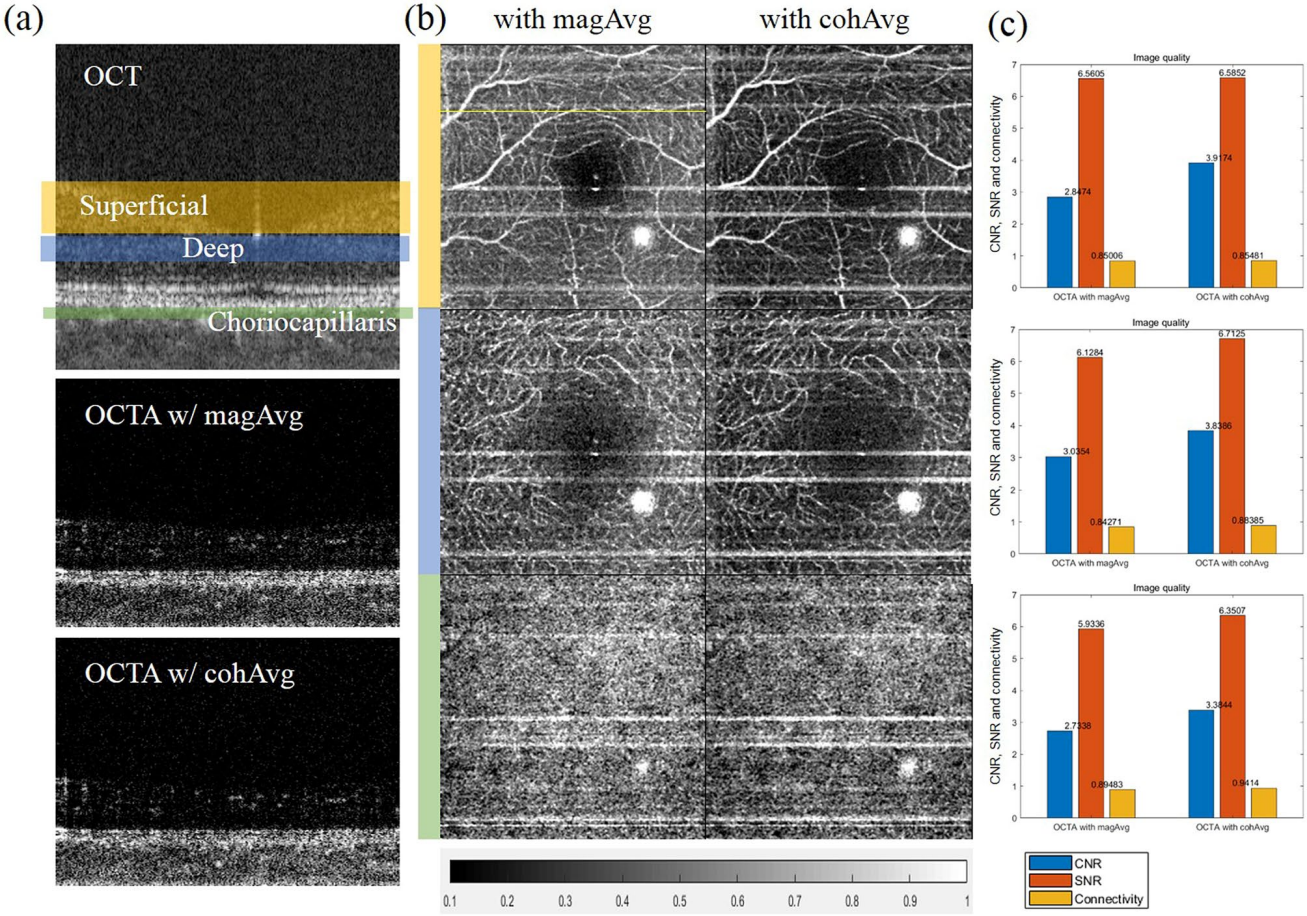
Assessment of en face OCTA images for human eye obtained by magnitude averaging (magAvg) or coherent averaging (cohAvg). (**a**) Cross-sectional OCT and OCTA images with magAvg and cohAvg. (**b**) En face maximum intensity projections (MIPs) over the specific depths (superficial, deep, choriocapillaris) of OCTA images. (**c**) Evaluations of the image quality (CNR, SNR, and connectivity) of images (**b**).

Next, we performed in vivo human retinal imaging with motion artifacts and assessed the quality of the acquired images. Figure 6a shows the acquired cross-sectional OCT and OCTA images. The en face MIPs of the cross-sectional OCTA images over the depth corresponding to the area are indicated in the OCT images of Fig. 6a, and the vascular network for each corresponding layer is shown in Fig. 6b. The cross-sectional OCTA image in Fig. 6a is from the location of the yellow line shown in Fig. 6b. Figure 6c shows the calculated CNR, SNR, and connectivity for each en face MIP. As was seen in the results of the phantom experiment, the OCTA image using cohAvg in the in vivo human retinal imaging experiment was superior to that with magAvg for CNR, SNR, and connectivity. However, despite motion correction, the horizontal white stripes that remained were thicker when cohAvg was applied. The evaluation indices for the OCTA images (CNR, SNR, and connectivity) are affected by the shapes of the blood vessels and reflectivity of the surrounding tissues; therefore, direct comparisons between the MIPs at different layers were impossible. However, when cohAvg was applied to the superficial layer, the CNR improved the most, and the degree of improvement decreased in the order of the deep and choriocapillaris layers. The CNRs of the layers of the human retina OCTA image to which cohAvg was applied were 3.9174, 3.8386, and 3.3844, respectively, which were higher than those of the OCTA image to which magAvg was applied, i.e., 2.8474, 3.0354, and 2.7338, respectively.

## Discussion

AML laser-based OCT systems can provide stable signals with small timing jitter, but the quality of the OCT images has been poor until recently owing to the roll-off characteristics. However, in our previous study, we developed an AML laser-based OCT imaging system with improved roll-off characteristics and showed the possibility of its application to ophthalmology via OCT images of the human retina. In this study, by taking advantage of the stable OCT signal obtained with the AML laser, the cohAvg method was introduced to obtain a contrast-improved OCTA image of the human retinal vasculature, which is essential in ophthalmology. The cohAvg method has previously been used to increase the SNRs of OCT images, but this is the first time that it has been applied to acquiring OCTA images with improved quality. In the experiment using the retina phantom, by comparing the contrast of the OCTA images for the cohAvg and magAvg methods, it was shown that applying cohAvg could increase the signal level in the OCTA image by approximately 3 dB (Fig. 4c,d). In this process, when applying the magAvg and cohAvg to a dynamic signal whose intensity changes over time, the magAvg signal level converges to its mean, and the cohAvg signal level converges to its minimum level (Fig. 3d). Thus, it was observed that OCTA by cohAvg had a larger variance than magAvg, thereby improving the contrast of the OCTA image. However, even in the phantom experiments, additional motion artifacts were not excluded; this reduced the degree of contrast enhancement by cohAvg in the phantom experiment. In the in vivo human retinal imaging experiments involving motion artifacts, it was observed that the OCTA signal level in the TR increased and the contrast enhancement by cohAvg was correspondingly reduced (Fig. 6). In addition, the decrease in contrast enhancement at the deeper layer could be interpreted as a decrease in the dynamic range as the intensity of the flow signal weakens. Nevertheless, the applicability of cohAvg to an AML laser-based OCTA system with a stable phase seems sufficient to improve the contrast of the OCTA images. Furthermore, developing an AML laser with a high sweep rate or a novel motion compensation algorithm will help maximize the effects of cohAvg. Thus, an AML laser that provides stable OCT signals will be useful for systems such as polarization-sensitive OCT or optical coherence elastography that use phase measurements.

Unlike SS-OCT, spectral-domain OCT (SD-OCT) uses a system where the phase is intrinsically stable. This is advantageous for phase-based OCT applications such as PS-OCT or OCTA. The phase stability of the akinetic swept-source AML laser proposed in this study is similar to that of existing SD-OCT. Therefore, it is expected that the CNR improvement method of OCTA using coherent averaging introduced in this study can be applied directly to the SD-OCT system without addition of an auxiliary interferometer for phase tracking.

In the present study, it was found that applying cohAvg to OCTA increased the sensitivity to flow signal in OCTA by 3 dB, thereby increasing the dynamic range. In the en face view OCTA images of the human retina, the CNR was enhanced by a factor of approximately 1.3. This improvement was due to the increase in the intensity of the dynamic (flow) signal compared to the static (tissue) signal. OCTA images with improved CNR, showing the flow signal clearly by separating it from the static signal, will help clinicians in the field make diagnostic judgments. However, because the intensity-based OCTA algorithm was used to determine the cohAvg effect, more research is needed on complex-based algorithms that are more sensitive to motion artifacts. These investigations will be undertaken in the future.

## Methods

### OCTA with coherent averaging

**Figure 7:**
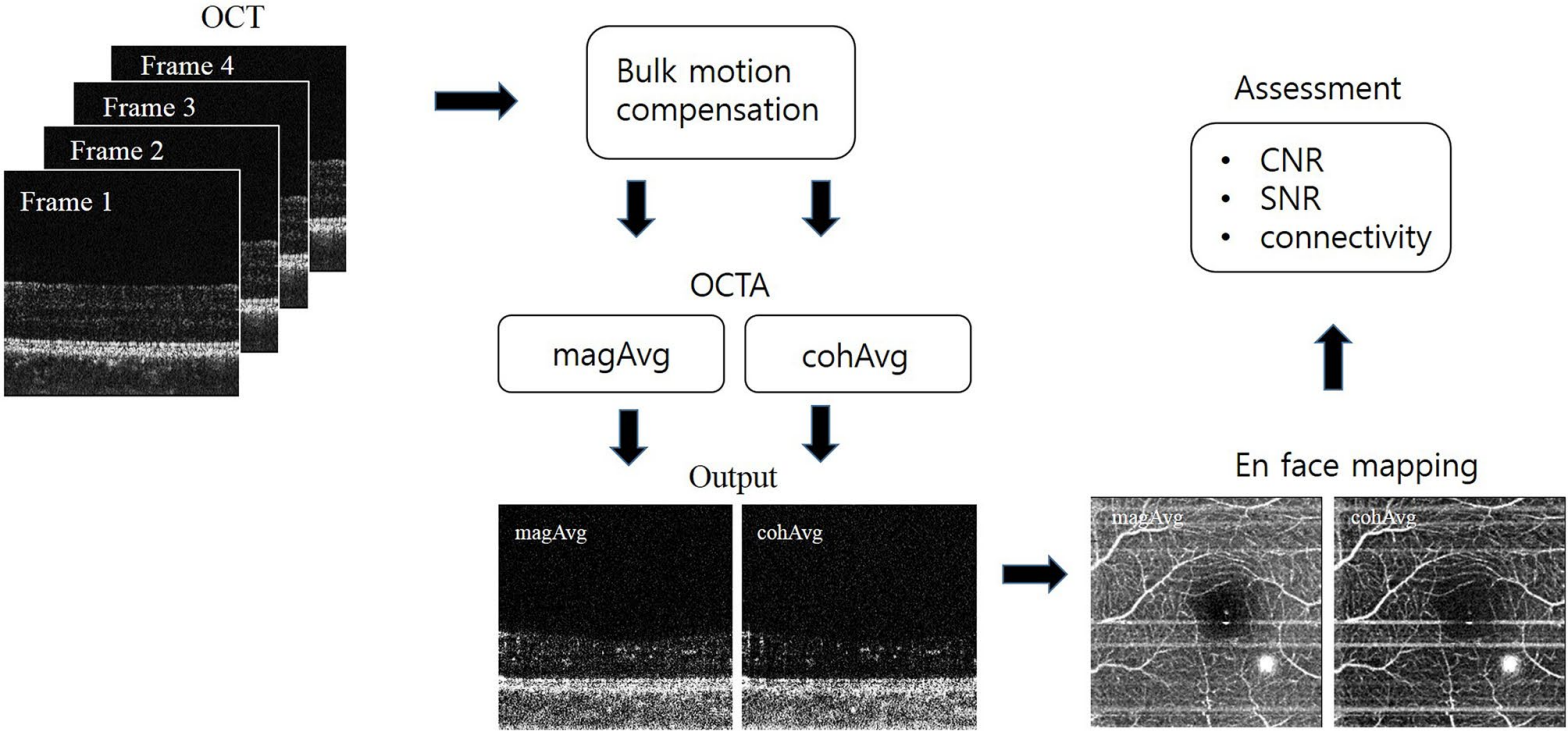
OCTA image acquisition procedure showing the differences between magAvg and cohAvg.

The OCTA algorithm used in the experiments was a speckle variance algorithm. The speckle variance algorithm extracts blood flow signals through the variance of amplitude fluctuations caused by blood flow. Therefore, the phase change caused by blood flow is ignored in the speckle variance algorithm. In this study, the general speckle variance algorithm was named OCTA with magAvg. However, the speckle variance algorithm that does not ignore phase change was named OCTA with cohAvg. Figure 7 shows the OCTA acquisition procedure to depict the differences between magAvg and cohAvg. First, bulk motion compensation was performed on four B-scan images acquired at different times in the same C-scan location, as shown in Fig. 2. Subsequently, Eq. (3) was used to obtain the OCTA with magAvg and cohAvg. Thereafter, the MIP image was acquired, and the CNR, SNR, and connectivity of the en face image were calculated.

### Quantification of en face OCTA images

To evaluate the quality of the en face OCTA images acquired by different algorithms, the CNR, SNR, and connectivity were assessed. Before calculating the CNR and SNR, the en face OCTA images were first normalized and converted to binary images by applying a threshold to separate the blood vessels from static tissues. Then, a skeleton map with a pixel width of 1 was generated from the binary image. The CNR and SNR of the image, where the value equals one in the skeleton map, M (x, y) = 1, is defined as.

4 $${\mathrm{CNR}}_{en\, face \,OCTA}= \frac{\overline{S\left(x,y\right){|}_{M\left(x,y\right)==1}}-\overline{S\left(x,y\right){|}_{Bck}}}{{\sigma }_{S(x,y)}{|}_{Bck}},$$

5 $${\mathrm{SNR}}_{en \,face \,OCTA} = \frac{\overline{S\left(x,y\right){|}_{M\left(x,y\right)==1}}}{{\sigma }_{S(x,y)}{|}_{Bck}},$$

where $\overline{S\left(x,y\right){|}_{M\left(x,y\right)==1}}$ is the mean value of the intensities of the pixels that match the skeleton map in the en face OCTA image; $\overline{S\left(x,y\right){|}_{Bck}}$ is the mean value of pixels corresponding to the background (tissue) area in the en face OCTA image, and ${\sigma }_{S(x,y)}{|}_{Bck}$ is the STD of the background signals. The connectivity is defined by

6 $$\mathrm{Connectivity}= \frac{{N}_{connected}{|}_{M\left(x,y\right)==1}}{{N}_{total}{|}_{M\left(x,y\right)==1}},$$

where ${N}_{connected}{|}_{M\left(x,y\right)==1}$ and ${N}_{total}{|}_{M\left(x,y\right)==1}$ are the number of connected pixels and the total number of nonzero pixels in the skeleton map, respectively.

### AML laser-based swept-source OCTA system and image acquisition

The configuration of the AML laser-based SS-OCT system used in the experiments is shown in Supplementary Figure S2. The AML laser, which was upgraded from a previous study, was used as the light source in the system^15^. The sweep rate of the laser was set to 100 kHz over a wavelength range of approximately 100 nm, from 1030 to 1130 nm, with a duty cycle of 90%. (In our previous study, the swept-wavelength range was only 80 nm; widening it enhanced the axial resolution^15^.) The beam from the AML laser was divided into a sample arm and a reference arm using a 50:50 fiber coupler. A 2-D galvanometric scanner was installed in the sample arm to enable 2-D scanning, and the two lenses were aligned in a telecentric design in front of the eye such that the collimated beam could be applied to the pupil. In addition, the focal plane within the sample was finely adjusted by an electric tunable lens (Arctic 39N1, Corning Varioptic) installed between the fiber collimator (FC) and 2-D galvanometric scanner to focus light accurately on the retinal surface. Optical alignment was performed at the reference arm so that the beam from the FC could be reflected to the FC, and a dispersion compensation plate (LSM05DC, Thorlabs) was installed to match the optical path length and dispersion with the sample arm. The beams returned from the sample and reference arms were recombined using the 50:50 fiber coupler, and the interference light was again divided in two and applied to the (+) and (−) ports of the balanced photodetector (BPD, 1817-FC, New Focus). After the direct current component was filtered by the BPD, only the alternating current component of the interference fringe signal was recorded, using a digitizer (ATS9350, AlazarTech) installed in the computer. Each A-scan signal was digitized into 2048 points at 250 MS/s. Thus, it took 2 ms to produce a B-scan and 2.5 s to produce an en face image ($250\times$ 4 $\times$ 250) with our system.

The synchronization among acquisition devices were shown in Supplementary Fig. S3. The sweep trigger from the AML laser and the arbitrary mask of the square-wave signal from a DAQ (data acquisition) terminal block were combined through a logical AND gate in a trigger synchronization box (Supplementary Fig. S3(a)). The synchronized master trigger was fed to the digitizer, thus minimizing the timing jitter between the galvanometer scanner (6210H XY Sets-3 mm, Cambridge Technology) and digitizer. At the same time, the master trigger was fed to the counter. As the master trigger was generated for each A-line scan acquired, the counter determined when to move to the next B-scan by checking the number of signal sets acquired in the current frame (Supplementary Fig. S3(b)). Four repeated B-scans were performed at the same C-scan location to obtain OCTA images according to the step bidirectional profile^19^, and the size of the volumetric data for the OCT/OCTA images was 2048(A-scan)$\times$ 250(B-scan)$\times$ 4(repeat)$\times$ 250(C-scan).

### Retina phantom

In a previous study, the production process of the retinal phantom was described in five steps^20^. Briefly, the retinal phantom consisted of multilayered thin films and microfluidic channels made of polydimethylsiloxane (PDMS) and titanium dioxide (TiO_2_) powder. In Step 1, PDMS was mixed with TiO_2_ particles using a probe tip sonicator. The concentration of TiO_2_ particles in the PDMS was adjusted to match the intensity of the retinal cross-sectional OCT images. In Step 2, the PDMS mixture was spin coated onto a glass substrate to form a multilayered thin film. The thickness of the layer was adjusted by adjusting the rotational speed and spin time of the spin coater. The PDMS mixture was cured on a hot plate after spinning, and the uncured PDMS mixture was then deposited over the cured thin film as the next layer. The spin-coating and curing processes were repeated twice. Therefore, the multilayered thin films had three layers. In Step 3, the two microfluidic channels were designed to mimic superficial and deep retinal vessels. The microfluidic channels were patterned in two silicon wafers using a photolithographic process, and these patterned wafers were used as molds for the microchannel fabrication. The PDMS mixture was poured into each patterned wafer and pressed with a glass plate. After curing, the microfluidic channel layers were detached from the wafer and glass. In Step 4, the multilayered thin film was positioned between the two microfluidic channel layers. All layers were attached to the oxygen plasma. Finally, the inlet and outlet of the retinal phantom were connected to the fluorinated ethylene propylene tubes. The fabricated retinal phantom was packaged with a lens in a cylindrical housing.

### In vivo human retinal imaging

This study was approved by the Institutional Review Board of Gwangju Institute of Science and Technology (Approval No. 20180629-HR-36-02-02). Written informed consent was obtained from a healthy subject before conducting the procedure. All experiments were performed in accordance with relevant guidelines and regulations. The subject was positioned on a commercial OCT headrest to obtain human retina OCT and OCTA images. The average laser power applied to the eye was limited to less than 1.5 mW, which is less than the ANSI limitation of 1.925 mW at 1060 nm.

## Supplementary Information

Supplementary Figures.
