# Supplementary material for: Phase stable swept-source optical coherence tomography with active mode-locking laser for contrast enhancements of retinal angiography
Source: Sci Rep. 2021 Aug 17;11:16636. doi: 10.1038/s41598-021-95982-9 (PMC8371173; doi:10.1038/s41598-021-95982-9)
Supplement: Supplementary file 1 — Supplementary Figures. [file 41598_2021_95982_MOESM1_ESM.pdf]

Phase stable swept-source optical coherence tomography with active mode-locking laser for contrast enhancements of retinal angiography

Kwan Seob Park[1], Eunwoo Park[1], Hwidon Lee[2,3], Hyun-Ji Lee[4,5], Sang-Won Lee[4,5], Tae Joong Eom[1,*]

[1]Advanced Photonics Research Institute, Gwangju Institute of Science and Technology, 123 Cheomdan-gwagiro, Buk-gu, Gwangju 61005, South Korea
[2]Harvard Medical School, Boston, Massachusetts 02115, USA
[3]Wellman Center for Photomedicine, Harvard Medical School and Massachusetts General Hospital, 40 Blossom Street, Boston, Massachusetts 02114, USA
[4]Safety Measurement Institute, Korea Research Institute of Standards and Science, 267 Gajeong-ro, Yuseong-gu, Daejeon 34113, South Korea
[5]Department of Medical Physics, University of Science and Technology, 217 Gajeong-ro, Yuseong-gu, Daejeon 34113, South Korea

[*]Correspondence and requests for materials should be addressed to T.J.E. (eomtj@gist.ac.kr)

Supplementary Figures

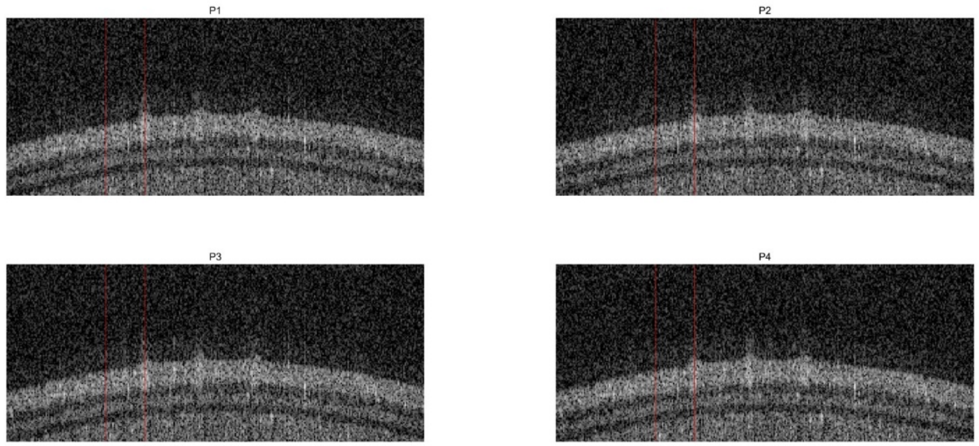

Supplementary Figure S1 | Four-consecutive B-scans(P1-4) at the same C-scan location.

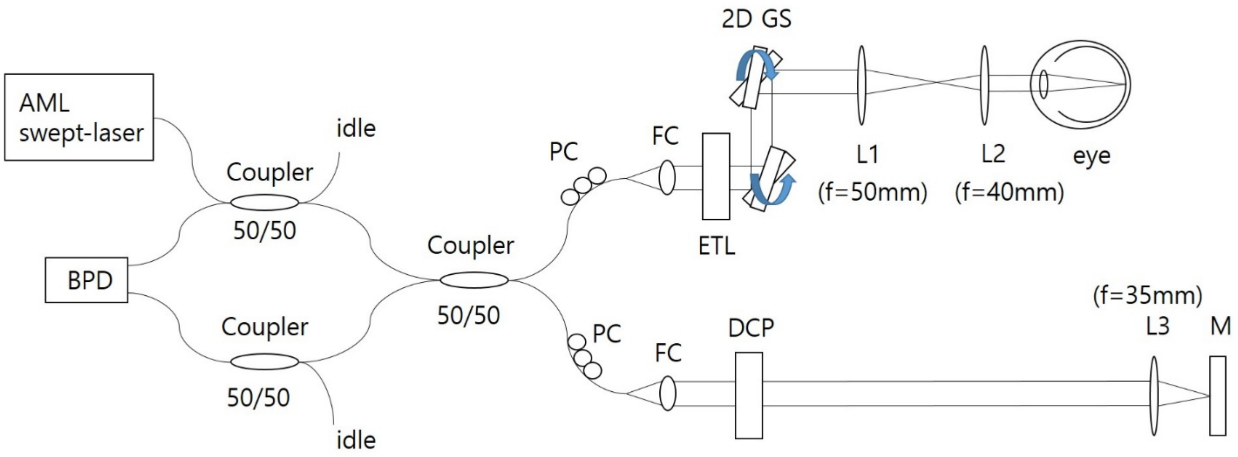

Supplementary Figure S2 | Illustration of experimental setup.
AML: active-mode locking laser, BPD: balanced photodetector (1817-FC, New Focus), PC: polarization controller, FC: fiber collimator, 2D GS: 2 dimensional galvanometric scanner (6210H XY Sets-3mm, Cambridge Technology), DCP: dispersion compensation plate (LSM05DC, Thorlabs), ETL: electric tunable lens (A-39N-1, Corning Varioptic), L1~3: lens, M: mirror.

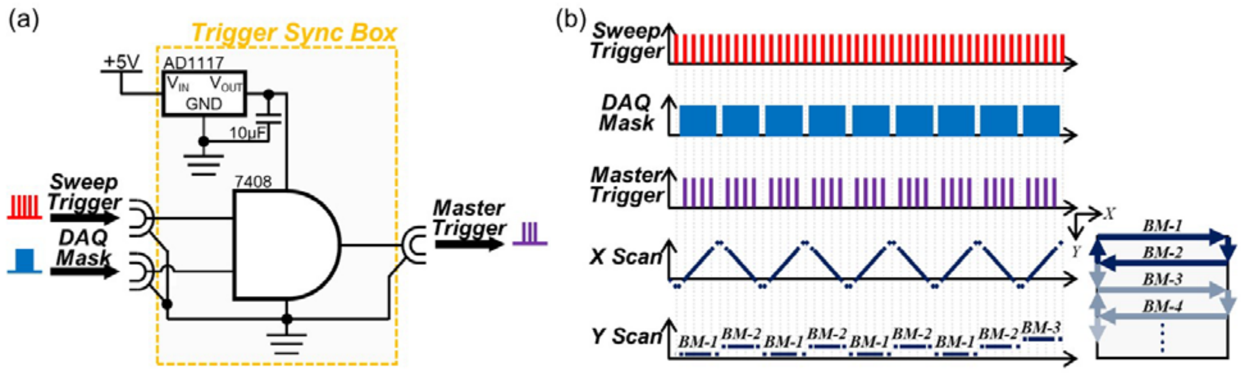

Supplementary Figure S3 | Signal synchronization in OCTA.
(a) Schematic of the trigger synchronization box. (b) Synchronization timing diagram for data acquisition.
